# Predicting first-time anaphylaxis in the elderly using stacked machine learning and population registers

**DOI:** 10.3389/falgy.2025.1655662

**Published:** 2025-11-06

**Authors:** Toni Mora, David Roche, Rosa Muñoz-Cano

**Affiliations:** 1 Research Institute for Evaluation and Public Policies (IRAPP), Universitat Internacional de Catalunya (UIC), Barcelona, Spain; 2 Allergy Department, Hospital Clinic, Barcelona, Spain; 3 MeTRI^2^A—Institut d’Investigacions Biomèdiques August Pi I Sunyer (IDIBAPS), Barcelona, Spain; 4 RICORS—REI-Instituto de Salud Carlos III, Madrid, Spain

**Keywords:** anaphylaxis prediction, stacked machine learning model, administrative healthcare data, elderly population, healthcare utilisation patterns, artificial intelligence, allergy risk stratification

## Abstract

**Background:**

Anaphylaxis is a severe, potentially life-threatening allergic reaction that requires rapid identification and intervention. Predicting individuals at risk remains a clinical challenge due to its multifactorial nature and variable presentation.

**Objective:**

To develop and evaluate explainable machine learning models that predict the risk of anaphylaxis using routinely collected clinical data.

**Methods:**

We analysed a matched case-control dataset derived from anonymised electronic health records. After applying chi-squared-based feature selection, we trained multiple classification algorithms—including logistic regression, decision trees, random forests, XGBoost, and a stacking ensemble. Model performance was evaluated using AUC, sensitivity, specificity, precision, and F1-score. SHAP values were used to assess model explainability.

**Results:**

The best-performing model achieved an AUC of 0.79, demonstrating high discrimination and balanced sensitivity/specificity. Key predictors included healthcare utilisation patterns, age, socioeconomic proxy (copayment level), and specific diagnostic codes related to allergic conditions.

**Conclusion:**

This study demonstrates the potential of interpretable machine learning approaches to support the early identification of individuals at high risk of anaphylaxis. These tools can enhance clinical risk stratification and inform preventive strategies in routine practice.

## Introduction

Anaphylaxis is a severe and potentially life-threatening allergic reaction that can occur rapidly and demands immediate medical intervention. Despite its clinical urgency, predicting the onset of first-time anaphylaxis (the first recorded diagnosis of anaphylaxis) remains a significant challenge. This difficulty arises from the complex interplay of individual risk factors, including underlying health conditions, polypharmacy, and socioeconomic determinants, which are often not adequately captured in current screening protocols (1, 2).

In middle-aged and elderly populations, anaphylaxis is often more severe and associated with higher hospitalisation rates compared to younger groups. Insect venoms and medications, particularly analgesics and antibiotics, are the predominant triggers, while cardiovascular comorbidities contribute to more serious clinical presentations such as hypotension or syncope (3–8). Despite this elevated risk, adrenaline remains underutilised, with administration reported in only about 30% of elderly cases. Furthermore, atypical presentations and symptom overlap with comorbidities contribute to frequent underdiagnosis and mismanagement (2, 3, 9). These challenges underscore the urgent need for age-specific diagnostic criteria, treatment protocols, and improved education for healthcare professionals to ensure timely recognition and management in emergency settings.

Improving awareness of the unique clinical profile of anaphylaxis in older adults and integrating geriatric considerations into clinical guidelines are essential steps toward more effective prevention and management strategies. Advancements in Machine Learning (ML) provide promising solutions for identifying individuals at high risk by analysing large-scale healthcare datasets (10). ML models can uncover hidden patterns and complex relationships within extensive and multifaceted data, enabling accurate predictions that can inform early intervention strategies. In the Spanish national health system, drug co-payment levels (TSI categories) are defined by income and pension status. Although not a direct clinical factor, these categories provide a standardised proxy for socioeconomic status, which may influence both healthcare access and the likelihood of anaphylaxis being diagnosed and treated. These administrative data, while originally collected for billing and management purposes, also reflect patterns of access, socioeconomic disparities (through drug co-payment levels), and healthcare utilisation, which are directly relevant for identifying vulnerable patients and improving care pathways in anaphylaxis. The integration of machine learning and artificial intelligence into allergy care offers promising avenues for improving early risk detection and enabling personalised management strategies.

This study aims to develop a robust predictive model for first-time anaphylaxis diagnoses in the elderly using an ensemble machine-learning approach. By combining multiple machine learning models, the ensemble strategy enhances predictive accuracy and reliability, capitalising on the strengths of different algorithms (11). The analysis leverages a comprehensive administrative healthcare dataset from Catalonia, encompassing nearly 3 million elderly individuals between 2014 and 2021.

Key predictive factors in the model include the frequency and variability of healthcare visits, specific medical diagnoses, and socioeconomic indicators such as drug co-payment levels. This model aims to support healthcare professionals and policymakers in implementing targeted prevention strategies and optimising healthcare resource allocation by identifying patterns associated with increased anaphylaxis risk. As highlighted by Indolfi et al. (12), artificial intelligence has strong potential to enhance allergy management throughout the lifespan, from childhood to adulthood, especially by improving early detection and enabling stratified care. Ultimately, this research contributes to advancing proactive and data-driven healthcare management, aiming to improve outcomes for vulnerable populations at risk of severe allergic reactions.

Recent bibliometric analyses have highlighted the growing interest in artificial intelligence applications across allergy and immunology, underscoring both emerging opportunities and persistent challenges in the field (13).

## Materials & methods

### Data collection and study cohort

This study utilises an extensive administrative dataset provided by the Agency for Health Quality and Assessment of Catalonia (AQuAS), incorporating healthcare information from multiple providers across various timeframes. The dataset focuses on the Catalan population born before January 1, 1965, forming a retrospective cohort of 2,924,590 individuals. That is, we considered those individuals who reached the age of 60 at any time over the considered period. Data spans from January 2014 to October 2021, encompassing records from primary care, hospitalisations, and emergency services. Each record includes a unique patient identifier, visit dates, age, primary and secondary diagnoses, and medical procedures. Additionally, these records are linked to demographic variables, including gender, birth date, drug co-payment level (as a proxy for socioeconomic status), nationality, date of death, and the individual's assigned healthcare region.

All records coded in ICD-9 up to 2017 were converted to ICD-10 to standardise diagnostic information. A patient was classified as an anaphylaxis case if at least one medical visit was coded with specific ICD-10-CM codes for anaphylaxis, including T78.0, T78.2, T80.5, and T88.6.

To enhance comparability between groups, we implemented a coarsened exact matching (CEM) algorithm to refine the selection of the control group. This matching method minimises the imbalance between cases and controls, ensuring more accurate comparisons. The resulting dataset comprised 8,250 individuals, of whom 4,051 had experienced at least one anaphylaxis diagnosis during the study period. This reflects a 1.22% prevalence rate of anaphylactic episodes in the elderly population.

### Model configuration and data preparation

The primary outcome of this study was the first-time diagnosis of anaphylaxis, framed as a classification task due to its binary nature. Each patient in the dataset was represented as a single observation, incorporating demographic attributes such as sex, socioeconomic status, co-payment level, nationality, and healthcare region. Additionally, all diagnoses and medical procedures unrelated to anaphylaxis were retained to capture the broader clinical context. Anaphylaxis was recorded using a dichotomous variable (1 for diagnosis, 0 for no diagnosis).

Following the methodology of Roche, Mora, and Cid (14), we structured the dataset so that each row contained patient identifiers, recorded medical visits (including diagnoses and procedures), and sociodemographic variables. Two new variables were generated for everyone: the total number of healthcare visits and the standard deviation of visit dates. These variables offer insights into healthcare utilisation patterns.

To refine feature selection, we created dummy variables for drug consumption using the Anatomical Therapeutic Chemical (ATC3) classification, which organises substances into chemical, pharmacological, or therapeutic categories. Age was treated as a continuous variable to preserve data granularity. We applied Lasso regression and frequency encoding for diagnosis and procedure variables to minimise noise and irrelevant data that could hinder model accuracy.

The dataset initially contained 9,803 dichotomous variables, covering 6,842 diagnoses, 2,363 medical procedures, 69 ATC3 drug categories, and 522 healthcare providers. We employed a Chi-square test for feature selection to reduce dimensionality and enhance performance. This test identified the most significant predictors by measuring the association between each feature and the target variable. This process narrowed the dataset to 200 highly relevant variables, optimising model performance.

All data preprocessing and structuring tasks were performed using Stata 18.0, while model computations and analysis were executed with Python 3.9.13.

### ML algorithms

After completing the data preprocessing phase, we implemented four machine learning algorithms to predict first-time anaphylaxis diagnoses: Logistic Regression (LR), Decision Tree (DT), Random Forest (RF), and Extreme Gradient Boosting (XGB). The dataset was divided into 80% for training and 20% for testing to evaluate model performance effectively. During the data transformation stage, all variables in the training set were standardised using z-score normalisation, ensuring that each feature had a mean of zero and a standard deviation of one. This standardisation was applied to the test set to maintain consistency and prevent data leakage.

We fine-tuned hyperparameters to optimise each model's performance using accuracy as the primary evaluation metric. This optimisation process involved conducting five-fold cross-validation within the training set, which systematically split the data into five subsets to ensure robust performance and prevent overfitting. Recognising the potential for improved accuracy, we then implemented a stacked ensemble learning approach. This method combined the predictive outputs of four distinct base learners (LR, DT, RF, and XGB), rather than multiple versions of a single model type. It utilised Logistic Regression as a meta-learner to aggregate the predictions, following best practices commonly cited in the literature (11). This ensemble strategy aimed to leverage the strengths of each model while compensating for its weaknesses.

We calculated standard classification performance metrics for model evaluation, including the Area Under the Curve (AUC), Accuracy, Precision, Specificity, Sensitivity, and the F1-Score. These metrics provided a comprehensive understanding of the model's predictive capabilities. Additionally, we assessed the importance of features to identify which variables had the most significant influence on the stacked model's predictions. This was achieved by ranking features according to their impact, with each model's contribution weighted by its coefficient within the final logistic regression meta-model. This approach ensured a balanced interpretation of how individual features across different algorithms influenced the overall model output.

## Results

Table 1 provides the descriptive statistics for the entire population, the matched sample, and the groups disaggregated by anaphylaxis diagnosis. Statistically significant differences were identified for variables typically associated with a higher prevalence of anaphylaxis, such as age, mortality rate, chronic comorbidities, nationality, and drug co-payment levels (15). The matched sample effectively reduced disparities across these variables, ensuring comparability between the groups. The average age of individuals diagnosed with anaphylaxis was marginally lower compared to those without it. At the same time, the presence of chronic comorbidities, as reflected in the AMG (Adjusted Morbidity Group) index, was notably higher in the anaphylaxis group. Although the proportion of females in the population was similar across groups, no significant gender-based differences were observed. Differences were observed across co-payment categories between groups.

**Table 1:** Descriptive statistics of the selected sample.

Variables	(1) No anaphylaxis (*N* = 2,792,764)	(2) No anaphylaxis matched (*N* = 4,199)	(3) anaphylaxis (*N* = 4,199)	(4) anaphylaxis prevalence
Average age	71.87	70.02	70.01*	–
% Passed away	0.17	0.17	0.16*	0.013854
Average AMG	13.02	17.88	18.28*	–
% Female	0.54	0.55	0.55	0.015213
Drug copayment levels (%)				
Exempted	19.10	20.05	20.26	0.0016136
10% copayment	53.71	54.68	54.27	0.0015371
40% copayment	14.42	14.22	14.29	0.0015071
50% copayment	9.92	8.98	9.02	0.0013846
60% copayment	1.14	1.02	1.08	0.0014411
Mutualists	1.71	1.05	1.08*	0.0009603
Nationality (%)				
Spain	95.33	96.59	95.89*	0.015301
Maghreb	0.88	0.64	0.78	0.0013337
South America	0.97	0.55	0.66**	0.0010290
East Europe	0.69	0.64	0.73	0.0016057
Europe	1.13	0.88	0.99	0.0013268
East Asia	0.22	0.19	0.19	0.0013038
South Asia	0.18	0.17	0.19	0.0016158
Central/Western Asia	0.13	0.02	0.02**	0.0002827
Central America/Caribbean	0.21	0.10	0.16	0.0011769
Middle West	0.09	0.05	0.12	0.0020602
North America	0.05	0.02	0.07	0.0019868
South-East Asia & Oceania	0.07	0.14	0.16*	0.0038398
East/Central/South Africa	0.02	0.00	0.02	0.0021459
No identified	0.03	0.00	0.02	0.0011261
Av. std dev. dates visits over rank period	79.49	68.32*	59.62*	–
Av. No of visits over rank period	1.002	1.002**	1.001*	–

* and ** represent statistical significance at 1% and 5%, respectively.

Columns (2) and (3) were compared statistically.

Table 1 further illustrates the age-based prevalence of anaphylaxis, revealing an apparent increase in prevalence among older age groups. This trend highlights the greater vulnerability of elderly individuals, likely due to cumulative health risks and the impact of comorbid conditions. Regarding nationality, small but statistically significant variations were detected for certain groups, including those from South America and Eastern Europe, which may reflect broader environmental, social, or genetic factors influencing the likelihood of anaphylactic episodes. Overall, the descriptive statistics emphasise the importance of considering demographic and clinical variables when analysing the prevalence of anaphylaxis in the elderly population. This analysis supports the development of predictive models to identify individuals at high risk and inform public health strategies.

**Figure 1:**
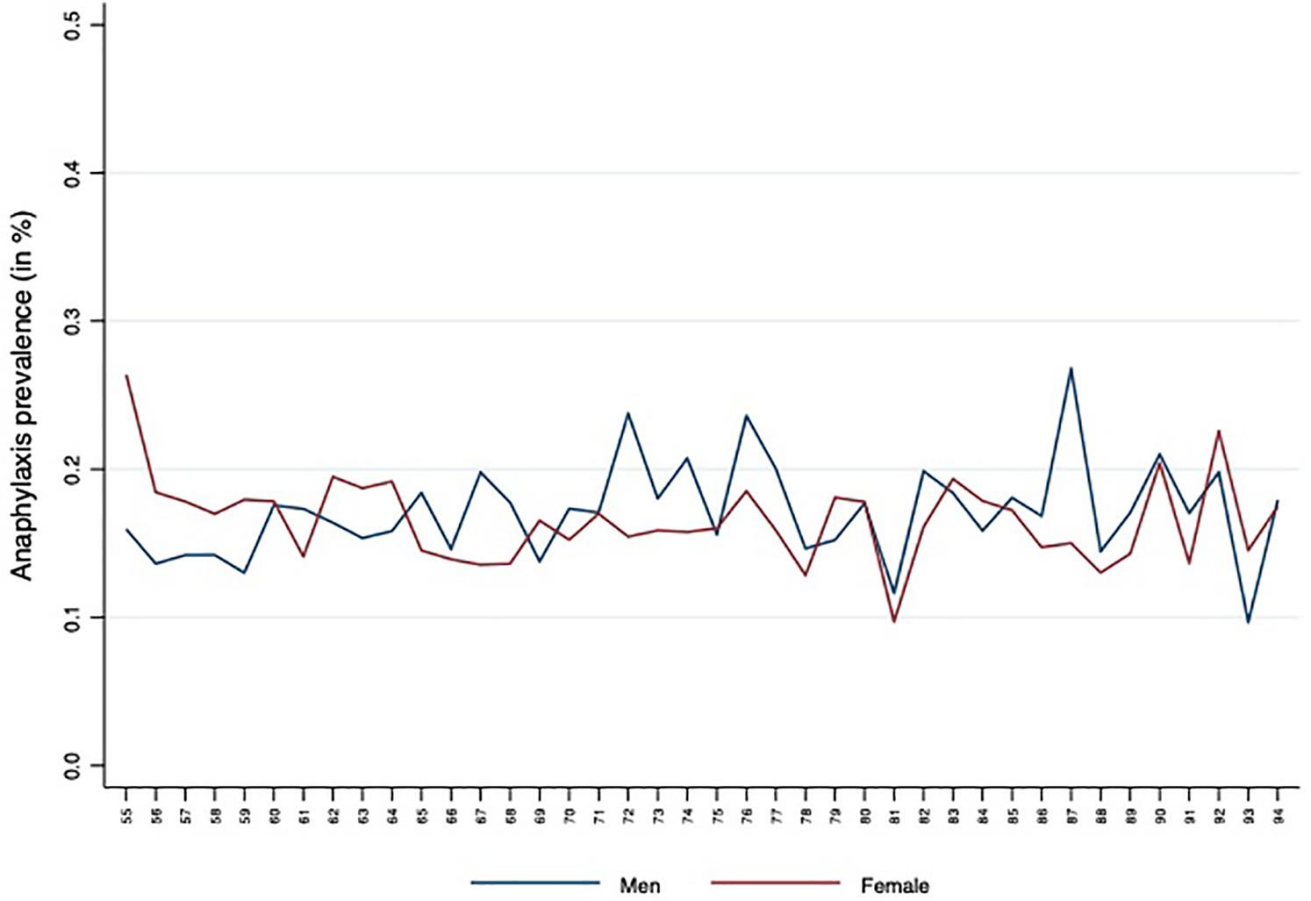
Anaphylaxis prevalence according to individuals' age. Prevalence of first-time anaphylaxis (%) across age groups, stratified by sex (men, blue line; women, red line).

Figure 1 illustrates the prevalence of anaphylaxis (in %) across different ages for men (blue line) and women (red line) in the elderly population. While both genders exhibit some fluctuations across age groups, no consistent sex-based pattern was identified. Age showed statistically significant associations with prevalence, but given the considerable sample size, even minor absolute differences reached significance. Overall, the prevalence of anaphylaxis remained low and relatively stable, with modest increases in older age groups. These findings suggest that while age-related variations exist, their clinical relevance should be interpreted with caution when assessing anaphylaxis risk in elderly populations.

**Figure 2:**
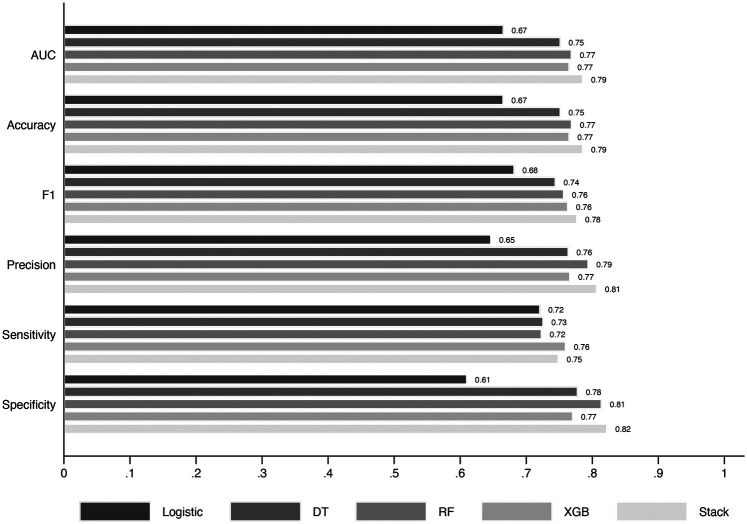
Performance metrics (AUC, accuracy, precision, sensitivity, specificity, F1-score) for logistic regression (LR), decision tree (DT), random forest (RF), extreme gradient boosting (XGB), and the stacked ensemble model. Confidence intervals for the stacked model are provided in the note. The CI for the stack figures was AUC (0.77–0.80), Accuracy (0.77–0.80), F1 (0.76–0.79), Precision (0.78–0.83), Sensitivity (0.72–0.77), and Specificity (0.80–0.85).

In the final stacked model (Figure 2), most performance metrics surpassed 74%, except for the Logistic Regression (LR) model, which consistently underperformed compared to other algorithms (16). Specifically, the stacked model achieved an accuracy of 78.6%, with a specificity of 82.3% and a sensitivity of 74.9%. The Area Under the Curve (AUC) score of 78.6% reflects a moderate yet robust predictive capacity, aligning well with the results of other performance indicators. Additionally, the model's F1-score, which balances precision and sensitivity, reached 80.7%, highlighting its effectiveness in managing the trade-off between correctly identifying true positives and minimising false positives.

As illustrated in Figure 2, the stacked model consistently outperformed individual models, such as Decision Tree (DT), Random Forest (RF), Extreme Gradient Boosting (XGB), and Logistic Regression (LR), across all evaluated metrics, particularly in terms of specificity and precision, where it demonstrated superior reliability. These results confirm that the ensemble approach significantly enhances the model's predictive performance, and the metrics obtained from the test set reinforce the model's strong generalisation capability (see Supplementary Table 1 for the stacked model's confusion matrix).

We subsequently calculated the contribution of each machine learning model within the stacked ensemble to evaluate its influence on the final prediction. The Random Forest (RF) algorithm made the most significant contribution to the overall performance of the models. In comparison, the contributions from Logistic Regression (LR), Decision Tree (DT), and Extreme Gradient Boosting (XGB) were 14.7%, 10.7%, and 12.3%, respectively. This distribution highlights the dominant role of RF in enhancing predictive accuracy within the stacked model.

**Figure 3:**
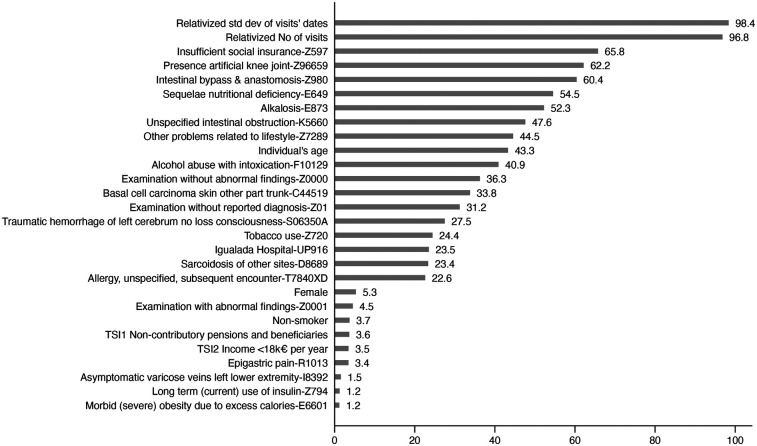
Variable importance rank for the stacked model. Variable importance ranking for the stacked model. Higher scores (0–100) indicate a greater absolute influence on model predictions, regardless of the directionality. The top predictors include healthcare utilisation patterns, diagnostic codes, and socioeconomic indicators.

Figure 3 presents the ranking of variable importance, where higher values (up to a maximum of 100) indicate greater influence on the model's predictions. In this ranking, importance values are based on the absolute contribution of each variable to model predictions, meaning they capture the magnitude of influence irrespective of direction. The analysis identified four key factors as the most significant predictors of a first-time diagnosis of anaphylaxis in the elderly. The most influential variable was the standard deviation of visit dates, reflecting irregular patterns of healthcare utilisation. This was followed closely by the total number of healthcare visits, which suggests that frequent medical consultations may signal underlying health issues related to anaphylaxis risk. Whether a greater or smaller value of a given predictor increases risk is determined by the directionality of model coefficients or SHAP distributions (Supplementary Figure A1), rather than by Figure 3 itself.

Additionally, specific diagnoses, such as nutritional deficiencies (E649) and intestinal bypass procedures (Z690), emerged as critical predictors, highlighting possible health complications that could predispose individuals to severe allergic reactions. Another crucial factor was socioeconomic status, approximated through drug co-payment levels, which may indicate disparities in healthcare access and preventive care. Lastly, specific medical procedures, including the presence of artificial knee joints (Z9659), were also associated with a higher likelihood of anaphylaxis, potentially due to post-surgical complications or heightened sensitivity to medications.

These findings highlight the combined impact of healthcare utilisation patterns, underlying health conditions, and socioeconomic factors on predicting anaphylaxis risk. The stacked model effectively integrates these diverse predictors, improving its ability to identify high-risk individuals and supporting proactive healthcare interventions.

The SHAP (Shapley Additive explanations) summary plot illustrates the most influential features impacting the model's prediction of first-time anaphylaxis diagnoses (see Supplementary Figure A1). Variable importance was derived from the absolute mean SHAP values, reflecting overall influence irrespective of direction. Each point represents an individual data instance, with colour intensity reflecting feature values (blue for low and red for high values) and the *x*-axis showing the direction and magnitude of each feature's impact on the prediction. The most impactful predictor was the Relativised Number of Visits, where more healthcare visits strongly increased the likelihood of an anaphylaxis diagnosis. Similarly, the Relativised Standard Deviation of Visits' Dates was highly significant, suggesting that irregular healthcare visit patterns may be linked to increased risk.

Another critical predictor was the Examination without Abnormal Findings (Z0000) diagnosis, indicating that frequent check-ups without reported issues might still precede severe allergic events. Individuals' age also contributed significantly, with older individuals being more susceptible to anaphylaxis. Socioeconomic status, as captured by TSI1 Non-contributory Pensions and Beneficiaries and TSI2 Income <€18,000 per Year, showed that lower-income groups face higher risks, potentially due to disparities in access to preventive healthcare. Additional variables like Alcohol Abuse with Intoxication (F10129), Non-smoker status, and Tobacco Use (Z720) were also influential, although with a differential influence. Diagnoses such as Morbid (Severe) Obesity due to Excess Calories (E6601) and Epigastric Pain (R1013), as well as Allergy, unspecified, subsequent encounter (T7840XD), further contributed to the prediction, highlighting the role of underlying health conditions. While neither alcohol nor tobacco is a primary trigger of anaphylaxis, their presence may indirectly influence the severity and complexity of clinical presentations. In particular, cardiovascular comorbidities—frequently exacerbated by tobacco use—can heighten the risk of severe anaphylactic reactions in elderly individuals. Similarly, alcohol consumption may alter immune responses or interact with medications, potentially complicating management (17). These findings suggest that lifestyle factors, although not direct causes, should be considered in risk stratification and patient counselling, especially in older populations with multiple comorbidities.

## Discussion

A stacked machine learning model combining four predictive algorithms—Logistic Regression (LR), Decision Tree (DT), Random Forest (RF), and Extreme Gradient Boosting (XGB)—was developed to predict short-term, first-time diagnoses of anaphylaxis in the elderly. This model leveraged a comprehensive population-based administrative dataset from Catalonia, which included detailed information on demographics, socioeconomic status, medical diagnoses, prescribed medications, and medical procedures. By integrating these diverse data sources, the stacked model demonstrated superior performance compared to individual models, with the Random Forest (RF) algorithm making the most significant contribution to the final predictive outcome. The model achieved a promising AUC of 0.79, indicating a strong capacity to detect first-time anaphylaxis cases in the general population. These findings underscore the potential of explainable machine learning models to not only predict anaphylaxis risk but also to support clinical decision-making and optimise resource allocation in healthcare systems.

These findings also resonate with prior evidence highlighting clinical inertia in emergency care. Anaphylaxis is frequently underdiagnosed and, therefore, also undertreated (3, 8). Early identification through predictive models could improve recognition and reduce delays in epinephrine administration, ultimately improving outcomes, especially in elderly patients who are more likely to experience atypical symptoms (3, 4, 18). Presentations may mimic cardiovascular, respiratory, or gastrointestinal disorders, leading to diagnostic uncertainty and treatment delays. This is especially problematic in elderly patients, who are more likely to have comorbidities that obscure the clinical picture (3). Our findings underscore the importance of age-adapted clinical protocols and training to enhance the recognition of anaphylaxis in older adults. Our model, which incorporates healthcare utilisation patterns, comorbidity profiles, and socioeconomic indicators, offers a valuable tool to support early identification. By flagging individuals with irregular visit patterns, frequent consultations, or specific diagnostic histories, the model can prompt clinicians to consider anaphylaxis as a differential diagnosis even in ambiguous cases.

The model also highlights the importance of socioeconomic context. The association with drug co-payment levels suggests that socioeconomic inequalities may influence patterns of healthcare utilisation and diagnosis in anaphylaxis. As TSI categories are defined by income and pension status, they provide a proxy for socioeconomic position in the Catalan population. This finding should be interpreted cautiously, as co-payment is an indirect measure rather than a direct determinant of anaphylaxis risk. Drug co-payment levels, used as a proxy for income, were among the most influential predictors. While lower co-payment levels in Spain are associated with lower income, they may also reflect differences in health literacy or familiarity with navigating the healthcare system. These disparities could affect both exposure to allergens and access to timely care, reinforcing the need to integrate social determinants into clinical risk assessment.

Recent clinical perspectives have highlighted the urgent need to refine diagnostic techniques to improve early identification and reduce morbidity (19). ML models trained on historical patterns of utilisation and comorbidity profiles, such as the one presented in this study, could complement clinical judgement by flagging high-risk individuals based on historical data and prompt earlier suspicion in ambiguous cases (20). This approach could be particularly valuable in emergency departments, where time and information are limited. However, given the relatively low prevalence of anaphylaxis, immediate clinical application in such settings remains limited, and translation into practice should be regarded as a future goal. Further prospective studies and clinical validation are warranted to translate these models into real-world clinical tools, ensuring integration with electronic health records and acceptance by healthcare providers. It is worth noting that many of the top-ranked predictors identified, such as demographic or administrative indicators, are not directly modifiable at the clinical level. Others, such as healthcare utilisation patterns, may serve as proxies for broader underlying health status rather than actionable targets. Moreover, the dataset did not include information on perioperative exposures (e.g., antibiotic or analgesic use in patients with joint prostheses or intestinal anastomoses) or concomitant treatments, such as beta-blockers, which may also influence the risk of anaphylaxis. These limitations underscore that the variables highlighted by the model should be interpreted as markers of risk rather than causal factors.

Despite these positive results, several limitations must be acknowledged. First, the dataset exclusively covered individuals utilising public healthcare services in Catalonia, excluding those who rely solely on private healthcare providers. This limitation may restrict the model's generalisability, as healthcare access and utilisation patterns could differ across sectors. Second, the identification of anaphylaxis relied on direct diagnostic coding, which is known to underestimate true incidence in administrative datasets and may have led to conservative prevalence estimates. Third, the dataset lacked access to clinical biomarkers and environmental exposure data, which are known to influence allergic reactions and could enhance predictive accuracy. Fourth, while anaphylaxis events could be identified, information on their specific causes or triggers (e.g., food, drug, or venom) was not consistently available, preventing stratified analyses by aetiology. Fifth, the study period spanned the transition between ICD coding versions, and although diagnostic categories were harmonised for analysis, residual inconsistencies cannot be entirely excluded. Additionally, ICD-9 coding is less granular than ICD-10 for the diagnosis of anaphylaxis. Although ICD-9 records were harmonised to ICD-10 before analysis, this may have limited case ascertainment in the earlier study period (21). Sixth, the retrospective design may introduce biases related to evolving healthcare practices or changes in diagnostic coding.

In terms of clinical translation, our model should currently be regarded as an exploratory predictive framework rather than a deployable tool. Nonetheless, there are clear potential pathways for integration into practice. One option is embedding the model into electronic health records as a decision-support alert, flagging patients at higher risk to prompt clinician review. Alternatively, it could inform screening protocols in primary care, guiding referrals to allergy specialists or targeted preventive counselling. Importantly, some of the strongest predictors, such as healthcare utilisation and socioeconomic indicators, are indirect proxies that may reflect healthcare-seeking behaviour rather than underlying biological risk. This introduces potential bias, as frequent attenders may appear to be at higher risk independently of their true allergy propensity. Predictions should therefore be interpreted cautiously and always in conjunction with clinical judgement. Finally, although the dataset included drug consumption at the ATC3 category level, it lacked detail on specific prescription classes (e.g., beta-blockers, ACE inhibitors), as well as laboratory parameters, biomarkers, and measures of allergen exposure. The absence of these mechanistic variables constrains precision. Future iterations should therefore integrate richer clinical and biochemical datasets to enhance both accuracy and generalisability of risk estimation.

In conclusion, this study proves that machine learning models, particularly stacked ensemble methods, can effectively predict first-time anaphylaxis diagnoses using large-scale administrative healthcare data. To our knowledge, this is the first study to apply machine learning techniques for the early detection of anaphylaxis by analysing patients' historical diagnoses and medical procedures from administrative records. Beyond predictive accuracy, the model offers clinical value by supporting early identification, guiding targeted interventions, and informing resource allocation, and ultimately reducing morbidity and mortality associated with anaphylaxis in ageing populations. Future research should focus on incorporating data from private healthcare systems and integrating clinical biomarkers to improve prediction models further and support timely interventions for high-risk individuals.

## Data Availability

The original contributions presented in the study are included in the article/Supplementary Material, further inquiries can be directed to the corresponding author.

## References

[1] Muraro A Worm M Alviani C Cardona V DunnGalvin A Eigenmann P EAACI Guidelines: anaphylaxis (2021 update). Allergy. (2021) 76(12):3574–96. 10.1111/all.15032 34343358

[2] Khan BQ Lieberman P. Anaphylaxis in the elderly. Aging Health. (2008) 4(4):377–87. 10.2217/1745509X.4.4.377

[3] Aurich S Dölle-Bierke S Francuzik W Bilò MB Christoff G Fernandez-Rivas M Anaphylaxis in elderly patients—data from the European anaphylaxis registry. Front Immunol. (2019) 10:750. 10.3389/FIMMU.2019.00750 31068925 PMC6491699

[4] Ventura MT Boni E Taborda-Barata L Blain H Bousquet J. Anaphylaxis in elderly people. Curr Opin Allergy Clin Immunol. (2022) 22:435–40. 10.1097/ACI.0000000000000855 36165408 PMC10815006

[5] Campbell RL Hagan JB Li JT Vukov SC Kanthala AR Smith VD Anaphylaxis in emergency department patients 50 or 65 years or older. Ann Allergy Asthma Immunol. (2011) 106(5):401–6. 10.1016/J.ANAI.2011.01.011 21530872

[6] Rudders SA Banerji A Clark S Camargo CA Jr. Age-related differences in the clinical presentation of food-induced anaphylaxis. J Pediatr. (2011) 158(2):326–8. 10.1016/j.jpeds.2010.10.017 21094954 PMC3022088

[7] Meir LR Habbsa S Waqar O League C Li T Jongco AM. Anaphylaxis among elderly emergency department patients in a large health system in New York. Ann Allergy Asthma Immunol. (2022) 129(1):63–70.e3. 10.1016/j.anai.2022.03.020 35346881

[8] Arroyo AC Camargo CA. The importance of understanding anaphylaxis among older adults. Ann Allergy Asthma Immunol. (2022) 129(1):7–8. 10.1016/j.anai.2022.04.024 35717136 PMC9639602

[9] Ruiz Oropeza A Lassen A Halken S Bindslev-Jensen C Mortz CG. Anaphylaxis in an emergency care setting: a one-year prospective study in children and adults. Scand J Trauma Resusc Emerg Med. (2017) 25(1):111. 10.1186/s13049-017-0402-0 29166906 PMC5700668

[10] Beam A Kohane IS. Big data and machine learning in health care. JAMA. (2018) 319(13):1317–8. 10.1001/jama.2017.18391 29532063

[11] Zhou Z-H. Ensemble Methods: Foundations and Algorithms. Cambridge: Chapman and Hall/CRC (2012). ISBN: 978-1-4398-3003-1.

[12] Indolfi C Klain A Dinardo G Decimo F Miraglia del Giudice M. Artificial intelligence in the transition of allergy: a valuable tool from childhood to adulthood. Front Med (Lausanne). (2024) 11:1469161. 10.3389/fmed.2024.1469161 39219791 PMC11363185

[13] Xiao N Huang X Wu Y Li B Zang W Shinwari K Opportunities and challenges with artificial intelligence in allergy and immunology: a bibliometric study. Front Med (Lausanne). (2025) 12:1523902. 10.3389/fmed.2025.1523902 40270494 PMC12014590

[14] Roche D Mora T Cid J. Identifying non-adult attention-deficit/hyperactivity disorder individuals using a stacked machine learning algorithm with administrative data population registers in a universal healthcare system. JCPP Adv. (2023) 4:e12193. 10.1002/jcv2.12193 38486959 PMC10933630

[15] Nieto-Nieto A Tejedor-Alonso MA Farias-Aquino E Moro-Moro M Rosado Ingelmo A Gonzalez-Moreno A Clinical profile of patients with severe anaphylaxis hospitalized in the Spanish hospital system: 1997–2011. J Investig Allergol Clin Immunol. (2017) 27(2):111–26. 10.18176/jiaci.0146 28151396

[16] Gryak J Georgievska A Zhang J Najarian K Ravikumar R Sanders G Prediction of pediatric peanut oral food challenge outcomes using machine learning. J Allergy Clin Immunol Glob. (2024) 3(3):100252. 10.1016/j.jacig.2024.100252 38745865 PMC11090861

[17] Cook RT. Alcohol abuse, alcoholism, and damage to the immune system—a review. Alcohol Clin Exp Res. (1998) 22(9):1927–42. 10.1111/j.1530-0277.1998.tb03900.x 9884135

[18] Rossi CM Lenti MV Di Sabatino A. Adult anaphylaxis: a state-of-the-art review. Eur J Intern Med. (2022) 100:5–12. 10.1016/j.ejim.2022.03.003 35264295

[19] Wong DS Santos AF. The future of food allergy diagnosis. Front Allergy. (2024) 5:1456585. 10.3389/falgy.2024.1456585 39575109 PMC11578968

[20] Sutton RT Pincock D Baumgart DC Sadowski DC Fedorak RN Kroeker KI. An overview of clinical decision support systems: benefits, risks, and strategies for success. NPJ Digit Med. (2020) 3:17. 10.1038/s41746-020-0221-y 32047862 PMC7005290

[21] Eldredge CE Pracht E Gallagher J Tsalatsanis A. Direct versus indirect query performance of ICD-9/-10 coding to identify anaphylaxis. J Allergy Clin Immunol Pract. (2023) 11(4):1190–7.e2. 10.1016/j.jaip.2022.12.034 36621609

