# Supplementary material for: Predicting first-time anaphylaxis in the elderly using stacked machine learning and population registers
Source: Front Allergy. 2025 Nov 6;6:1655662. doi: 10.3389/falgy.2025.1655662 (PMC12631650; doi:10.3389/falgy.2025.1655662)
Supplement: Supplementary file 1 [file Supplementaryfile1.docx]

**Table A1. Confusion matrix for the stacked model**

|  | **Predicted False Anaphylaxis** | **Predicted True Anaphylaxis** |
| --- | --- | --- |
| **False** | 682 | 147 |
| **True** | 206 | 615 |

**Figure A1. SHAP summary plot showing feature contributions to anaphylaxis prediction.**

| 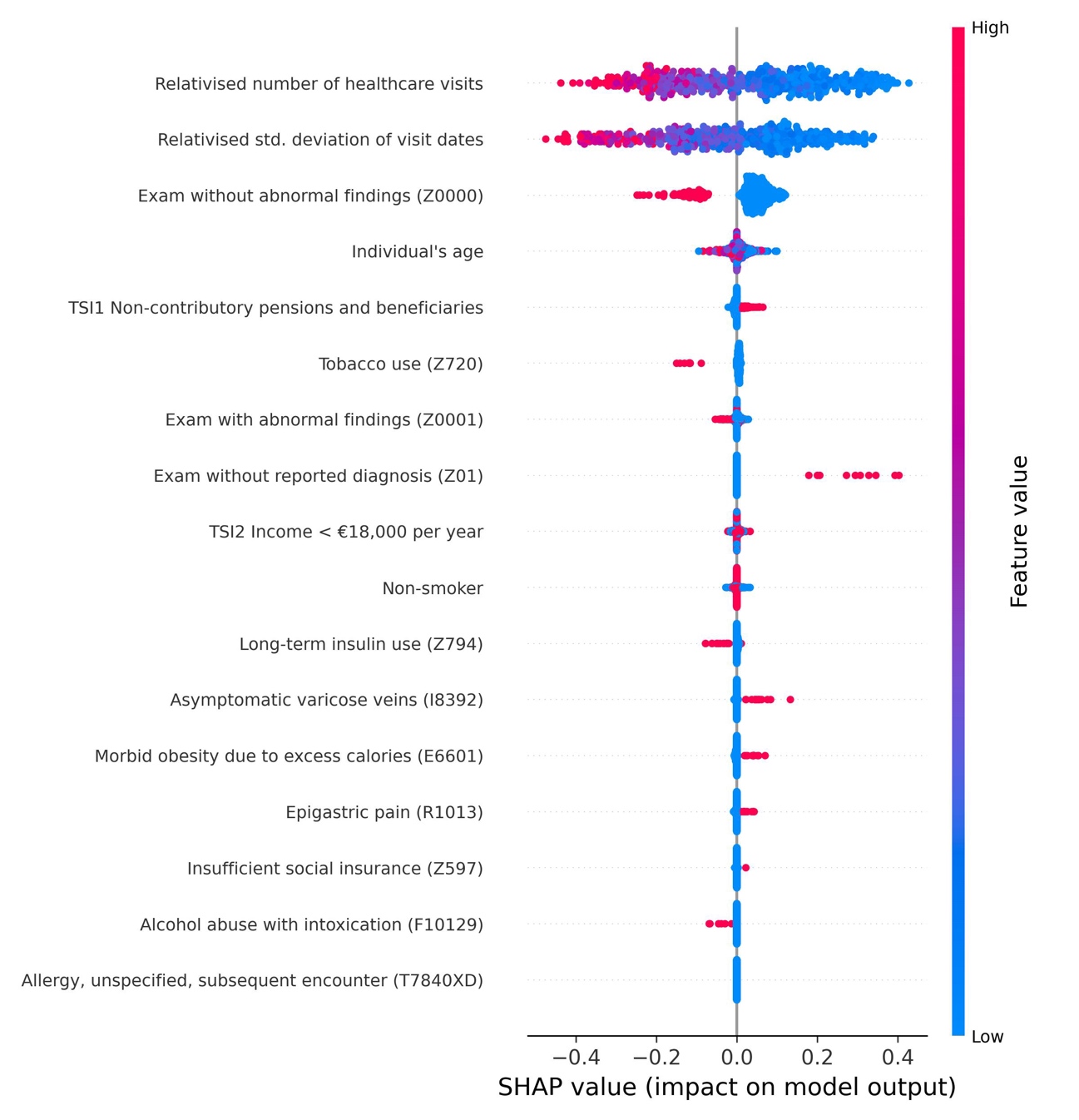 |
| --- |

Note: Each dot represents an individual patient. The position along the x-axis indicates the SHAP value, i.e., the feature's contribution to increasing (right) or decreasing (left) the predicted risk of anaphylaxis. Colours reflect the standardised value of each variable: red denotes high values and blue denotes low values. Variables are ordered by overall impact on the model, including clinical diagnoses, healthcare utilisation, and socioeconomic indicators.
